# Impaired Autonomic Responses to Emotional Stimuli in Autoimmune Limbic Encephalitis

**DOI:** 10.3389/fneur.2015.00250

**Published:** 2015-11-30

**Authors:** Olga Schröder, Elisabeth Schriewer, Kristin S. Golombeck, Julia Kürten, Hubertus Lohmann, Wolfram Schwindt, Heinz Wiendl, Maximilian Bruchmann, Nico Melzer, Thomas Straube

**Affiliations:** ^1^Institute of Medical Psychology and Systems Neuroscience, University of Muenster, Muenster, Germany; ^2^Department of Neurology, University of Muenster, Muenster, Germany; ^3^Department of Clinical Radiology, University of Muenster, Muenster, Germany

**Keywords:** amygdala, limbic encephalitis, emotion processing, dynamic stimulus material, skin conductance

## Abstract

Limbic encephalitis (LE) is an autoimmune-mediated disorder that affects structures of the limbic system, in particular, the amygdala. The amygdala constitutes a brain area substantial for processing of emotional, especially fear-related signals. The amygdala is also involved in neuroendocrine and autonomic functions, including skin conductance responses (SCRs) to emotionally arousing stimuli. This study investigates behavioral and autonomic responses to discrete emotion evoking and neutral film clips in a patient suffering from LE associated with contactin-associated protein-2 (CASPR2) antibodies as compared to a healthy control group. Results show a lack of SCRs in the patient while watching the film clips, with significant differences compared to healthy controls in the case of fear-inducing videos. There was no comparable impairment in behavioral data (emotion report, valence, and arousal ratings). The results point to a defective modulation of sympathetic responses during emotional stimulation in patients with LE, probably due to impaired functioning of the amygdala.

## Introduction

Limbic encephalitis (LE) refers to an adaptive neuron-directed and autoimmune-mediated inflammation with consecutive degeneration and structural reorganization within gray matter structures of the limbic system (1) characterized by epileptic seizures, progressive short-term memory deficits, as well as emotional and behavioral disturbance (2, 3). Magnetic resonance imaging (MRI) emphasizes this aberration by revealing changes in signal intensity and volume in regions within the medial temporal lobe, being mostly pronounced in the amygdala (4–7).

Evidence from animal and human research converges on the pivotal role the amygdala plays in emotion processing (e.g., experience and control of affect, emotional learning, emotional modulation of memory and attention, and regulation of emotion), this way contributing to appropriate homoeostatic behavior to emotionally salient stimuli (2, 8). Considering basic emotions, processing of negative emotions and especially of fear has been particularly disturbed by amygdala damage of various etiologies, including different forms of LE (9–15). However, some studies report apparently normal emotion processing in patients with amygdala damage, which might be due to the age at onset and the extent of the damage (16–18).

Beyond this, the amygdala has proved to be involved in neuroendocrine and autonomic functions, including skin conductance responses (SCRs) to emotionally arousing stimulation (19–22). Correspondingly, patients with right and bilateral amygdala damage are reported to show markedly reduced electrodermal responsiveness to emotional visual stimuli, which underscores the contribution of especially the right amygdala to the development of a general arousal level (23). Autonomic processing of arousing task demands in patients suffering from viral or autoimmune bilateral LE also revealed absent or strongly reduced sweat responses and skin vasoconstriction responses (24, 25).

Here, we report on an immunotherapy-naïve patient suffering from non-paraneoplastic autoimmune LE with contactin-associated protein-2 (CASPR2) antibodies at a very early stage of the disease (4 months after disease onset and 2 weeks after diagnosis). The study focuses on the investigation of how adaptive neuron-directed autoimmune inflammation in the early course of LE is reflected in behavioral and autonomic responses to discrete emotion evoking and neutral film clips as compared to performance in a neurotypical control group. These dynamic emotional stimuli were used in order to increase the complexity, intensity, and ecological validity of stimulus material (26). Six affective video clips were applied, and skin conductance (SC) was simultaneously recorded. Based on the assumed role of the amygdala in autonomic responses to emotional stimuli as it is outlined above, we hypothesized diminished reactivity of SC to emotional film clips, especially to fear-inducing videos, in the patient as compared to healthy controls.

## Materials and Methods

### Participants

#### Case Report

**Figure 1:**
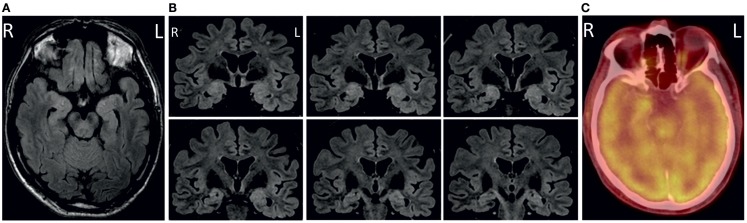
**Axial (A) and coronal (B) fluid-attenuated inversion recovery (FLAIR) images showing bilateral volume and signal increase in amygdala and anterior hippocampus**. Correspondingly, cerebral fluor-18-deoxyglucose-positron emission tomography/computed tomography (FDG-PET/-CT) revealed hypermetabolism of the right anterior mesial temporal lobe **(C)**.

We studied patient R.B., a 65-year-old left-handed male of Italian decent with lower secondary education (<9 years). R.B. suffered from LE associated with antibodies against CASPR2. He presented with progressive disturbance of attention and memory and a depressed mood since December 2013 (BDI-score: 31, severe depression). He experienced seven to eight nocturnal generalized tonic–clonic but no apparent complex partial seizures. Neurological examination was unremarkable. Medical history disclosed arterial hypertension, chronic obstructive pulmonary disease (COPD), and diabetes mellitus type II. He received oral treatment with valsartan 160 mg/day, hydrochlorothiazide 6.25 mg/day, and glimepiride 0.5 mg/day. HbA1_c_ was 7.1% (normal 4.3–6.1%) or 54.1 mmol/mol (normal 24–4 mmol/mol), the remaining routine laboratory findings were unremarkable. Peripheral nerve conduction studies were normal. Cerebral MRI at 3-T was performed at initial presentation in April 2013 and revealed bilateral volume and signal increase of amygdala and anterior hippocampus on axial and coronar T2-weighted and fluid-attenuated inversion recovery (FLAIR) sequences (Figure 1) consistent with temporo-mesial encephalitis (5, 6). Accordingly, cerebral fluor-18-deoxyglucose-positron emission tomography/computed tomography (FDG-PET/CT) at that time revealed hypermetabolism of the right anterior mesial temporal lobe (Figure 1). Cerebrospinal fluid analysis showed mild lymphomonocytic pleocytosis (7/μl; normal <5/μl) with mildly elevated protein levels (786 mg/l; normal <500 mg/l), and blood–cerebrospinal fluid barrier dysfunction (albumin ratio 11.5 × 10^−3^; normal <7.5 × 10^−3^) but no quantitative or qualitative evidence of intrathecal immunoglobulin synthesis. Glucose and lactate levels were normal. Anti-neuronal antibody testing in serum and cerebrospinal fluid showed high titers of antibodies against CASPR2 [titer 1:3200 in serum (normal <1:10) and 1:320 in cerebrospinal fluid (normal <1:10) on indirect immunofluorescence testing (IFT), Euroimmun Luebeck, Germany]. Mobile long-term surface electroencephalography recording for 3 days showed interictal anterior temporal epileptic activity, which was more pronounced on the right (85%) as compared to the left (15%) side. Moreover, two electroencephalographic seizures of right anterior temporal origin have been recorded. Clinical neuropsychological assessment revealed impairment of verbal but not figural learning and memory, attention, executive functions, and visuoconstruction (see Table 1 for detailed neuropsychological characteristics). A tumor search using whole-body FDG-PET/CT was unremarkable. R.B. received additional oral anticonvulsive treatment with levetiracetam (2 g/day); he was included in the study before initiation of any immunotherapy and more than 2 weeks after the last clinical seizure event.

**Table 1:** **Neuropsychological data**.

Test		PR	Interpretation
**Verbal memory functions**			
Verbal learning and memory test	Memory span (immediate recall, trial 1)	4	Far below average
	Learning performance (immediate recall, trial 5)	4	Far below average
	Free recall after distraction (trial 6)	4	Far below average
	Delayed free recall (trial 7)	4	Far below average
	Recognition (trial 8)	4	Far below average
**Visual memory functions**			
Rey–Osterrieth complex figure test	Immediate recall	18	Average
**Attention**			
Trail making test A	Processing speed	9	Far below average
**Executive functions**			
Wechsler memory scale, digits backward	Working memory	5	Far below average
Trail making test B	Cognitive flexibility	9	Far below average

*PR, percentile rank*.

#### Normal Controls

Nineteen healthy subjects (5 women and 14 men, mean age 32.9 ± 9.6 years, age range from 20 to 60 years) participated in the study as normal controls in exchange for monetary compensation. They were recruited via several public announcements. None of them reported any history of psychiatric and/or neurological disorders, treatment with psychotropic drugs or drug abuse or was under any medical treatment. All participants had normal or corrected-to-normal vision and were right handed, as determined by the Edinburgh Handedness Inventory (27). However, data of two participants were excluded from further analyses due to frequent talking during the videos, thus not guaranteeing full concentration on the stimuli. Data of one more control were expelled because she did not pass the respiration test (see Stimulus Material and Experimental Paradigm). This reduced the sample size to *n* = 16 [3 women and 13 men, mean age 30.2 ± 6.5 years, age range from 20 to 47 years, higher educational level (>12 years)].

#### Ethics Statement

All subjects gave written informed consent prior to participation. The study was approved by the local ethics committee (AZ: 2013-350-f-S, 2013-248-f-S).

### Stimulus Material and Experimental Paradigm

All participants were requested to watch six short film clips, each 40 s long, which were designed to elicit various emotional states (i.e., anger, sadness, disgust, fear, and happiness) and a non-emotional neutral state. The clips were excerpts from contemporary commercial movies and were applied without auditory content. They partially included scenes already itemized by other standardized batteries (26, 28, 29); other sequences were added by our group that were able to rapidly induce an emotional state, depicted potential real-life scenarios and were coherent in their features concerning the respective delineated emotion (30). The clips set was validated in a pre-study on an independent student sample (*n* = 51). Here, the subjects were asked to rate each of the film clips according to how much they experienced amusement, boredom, anger, sadness, fear, and disgust by using a 10-point Likert scale (0 = “not at all” to 9 = “most intensively”). Ratings of valence were obtained additionally (0 = “very pleasant” to 9 = “very unpleasant”). In order to assess the capacity of the film material to elicit various emotional states, a film (6 levels) × rating (6 levels) analysis of variance was conducted, with repeated measures on both factors (29). We found significant main effects of film (*F*_5,120_ = 42.78, *p* < 0.001, ϵ = 0.66, η^2^ = 0.47) and of rating (*F*_5,240_ = 14.69, *p* < 0.001, ϵ  = 0.68, η^2^  = 0.23) as well as a significant film × rating interaction (*F*_25,1200_ = 108.09, *p* < 0.001, ϵ = 0.42, η^2^ = 0.69) indicating differences in the rating profiles of the clips. *Post hoc* analyses were done using paired *t*-tests to compare the target emotion against the highest rated non-target emotion for each clip, with α-level Bonferroni-corrected for multiple comparisons (i.e., α = 0.05/6 = 0.0083). In all cases, target emotions were rated significantly higher. Table 2 presents a content description of the film set, along with mean scores of target emotion intensity and valence as well as detailed *post hoc* findings.

**Table 2:** **Description of the film clips**.

Film title	Target emotion	Content description	Reference	Intensity of target emotion	Valence	*Post hoc* analyses
Gran Torino	Anger	A boy is attacked by a gang of young men and is tortured by them	Eastwood (31)	*M* = 6.16, SD = 2.38	*M* = 7.16, SD = 1.70	Compared to fear: *t*_50_ = 4.31, *p* < 0.001
The Champ	Sadness	A little boy cries over his father’s dead body	Zeffirelli (32); Gross and Levenson (28); Hewig et al. (29); Rottenberg et al. (26)	*M* = 7.26, SD = 2.48	*M* = 6.78, SD = 1.81	Compared to fear: *t*_49_ = 15.01, *p* < 0.001
Christiane F. – we children from Bahnhof Zoo	Disgust	Two young adults sniff cocaine; the girl vomits	Edel (33)	*M* = 7.32, SD = 2.81	*M* = 7.88, SD = 1.69	Compared to sadness: *t*_49_ = 9.11, *p* < 0.001
Peas at 5:30	Fear	A blind man walks along the roof of a high rise building, impending to fall any time	Buechel (34)	*M* = 4.90, SD = 2.57	*M* = 5.78, SD = 1.20	Compared to boredom: *t*_50_ = 5.52, *p* < 0.001
Delicatessen	Happiness	A father brings out amazement in his two sons by showing them various tricks with soap bubbles	Jeunet and Caro (35)	*M* = 5.82, SD = 1.85	*M* = 2.18, SD = 1.42	Compared to boredom: *t*_49_ = 6.62, *p* < 0.001
All the President’s Men	Neutral	Two men have a conversation in a courtroom	Pakula (36); Hewig et al. (29)	*M* = 6.18, SD = 1.90	*M* = 4.25, SD = 1.46	Compared to amusement: *t*_50_ = 13.01, *p* < 0.001

*Scaling: valence (0 = “very pleasant,” 9 = “very unpleasant”), intensity (0 = “not at all intensive”, 9 = “most intensive”)*.

**M*, mean; SD, standard deviation*.

The film clips were presented on a conventional 27^′′^ computer screen with a resolution of 720 × 576, in a dimly lit room and under similar light conditions for every participant. The experimenter was present during the entire session. Before getting started, the participants were seated in front of the computer screen and were instructed to watch the emotion-eliciting film excerpts, each followed by three evaluation tasks. For the first, they should name an emotion that they had actually and primarily felt in response to viewing the respective film clip, while distinguishing this emotional state from their general mood state throughout the day and their idea what they should have felt during the film clip (37). The subjects had to choose from six options corresponding with the six emotional states the film set proved to cover. Following ratings of valence and arousal were conducted using the self-assessment manikin [SAM; (38)] on a nine-point Likert scale (from “1” to “9”) with higher values indicating more pleasant or more arousing emotional experiences. There was no time limit for the decision making, so that the experiment moved on only after pressing a button. The participants were explicitly reminded that there were no right or wrong answers in the experiment. A practice clip was shown in order to get accustomed to the rating procedure. After this, the experimenter positioned the SC sensors and conducted a respiration measurement during which subjects were asked to remain calm and inhale deeply several times. This was done in purpose of proving the general ability to develop SCRs. If so, the experiment was started. The interval between two film clips was set up at 10 s, during which subjects saw a fixation cross. Including the instructions, the whole experimental session took about 30 min.

### Skin Conductance Recording

Skin conductance activity was collected continuously and simultaneously during the presentation of the stimuli using a BIOPAC MP150 system and the corresponding software AcqKnowledge 4.3. (BIOPAC Systems, Inc.). Presentation software (Neurobehavioral Systems, Inc.) synchronized the physiological monitoring equipment by event markers designating the beginning and ending of each video. Ag/AgCl snap electrodes were used to receive the physiological signal at the thenar and hypothenar eminence of the non-dominant hand, thus allowing for a motor response performed by the dominant hand during the self-report tasks. The room temperature was kept similar for every participant (approximately 21°C).

In AcqKnowledge, a 0.5–2 Hz band-pass filter was applied. The number of thus extracted galvanic skin responses (nSCR) with trough-to-peak distance above 0.01 μS (39) provided a measure of SC activity for every film clip, in this manner reflecting phasic arousal responses over time of each kind of emotional stimulation (40, 41). Considering the typical latency of a SC response (1–3 s), our time window of interest was set to 1–40 s after stimulus onset.

Respiration test data were filtered analogously and were analyzed in terms of average trough-to-peak-distances.

### Data Analyses

Due to the nature of the data, results of emotional report are presented only descriptively. With regard to ratings of valence and arousal as well as data of physiological reactivity, an analytic approach reported by Franz et al. (42) was adapted to examine potential differences between R.B. and the control group. In this sense, R.B.’s values were *z*-transformed [*z* = (value_R.B._ − mean_controls_)/SD_controls_] to establish a relation to the standard distribution of the norm population (neurotypical control group). Thus, values of *z* = 0 conformed to the control group mean, *z* < 0 indicated lower and *z* > 0 expressed higher values. *Z*-values that were beyond the 95% confidence interval (i.e., |*z*| ≥ 1.96) of the control group were considered significantly abnormal.

## Results

### Subjective Emotional Responses

Due to technical failure of recording responses in two control measurements, emotional report data of only 14 control subjects could be considered in this section.

R.B. responded in accordance with intended inductions of specific emotion in case of anger, sadness, fear, and neutral film clips. By contrast, he described his emotional experience during watching the disgust eliciting film clip as fearful. The same response was given by one control subject as well, thus appearing an arguably possible emotional reaction to this film clip in a clinically healthy sample. Interestingly, though, R.B. characterized his emotional sensation during the happiness-inducing film clip as disgust. This response was not observed in any control subject. Full results are given in Table 3.

**Table 3:** **Naming the emotion induced by film clips**.

Emotion in the film	R.B.	Control group
Anger	Anger	Anger: 11/14 fear: 3/14
Sadness	Sadness	Sadness: 14/14
Disgust	Fear	Disgust: 13/14 fear: 1/14
Fear	Fear	Fear: 10/14 neutral: 4/14
Happiness	Disgust	Happiness: 12/14 neutral: 2/14
Neutral	Neutral	Neutral: 13/14 anger: 1/14

### Valence and Arousal Ratings

R.B.’s ratings of valence did not deviate significantly from the control group mean in cases of anger (*z*_anger_ = −0.30), sadness (*z*_sadness_ = −0.26), disgust (*z*_disgust_ = 0.26), and fear (*z*_fear_ = −1.09) eliciting films. R.B. tended to be more positive in the evaluation of the neutral film clip than the control group on overage (*z*_neutral_ = 1.94) although this difference was only marginally significant. R.B. assessed the happiness-inducing film clip as significantly more negative than it was averagely the case in the control group (*z*_happiness_ = −2.77).

R.B.’s ratings of arousal were not significantly different from the control group mean for happiness (*z*_happiness_ = −0.06), anger (*z*_anger_ = 0.66), disgust (*z*_disgust_ = −0.98), fear (*z*_fear_ = −1.08), and sadness-evoking film material (*z*_sadness_ = −1.81). The only exception was the neutral film clip: R.B. classified it as significantly more arousing than the control group on average (*z*_neutral_ = 2.07).

**Figure 2:**
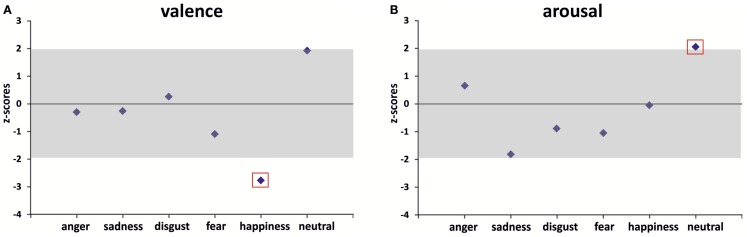
**R.B.’s *z*-transformed valence (A) and arousal (B) ratings vs. healthy controls (zero line represents control group’s mean; gray zone indicates the 95% confidence interval)**. Significant differences (i.e., |*z*| ≥ 1.96) are marked with a red box.

Results of both valence and arousal ratings are visualized in Figure 2.

### Physiological Reactivity

**Figure 3:**
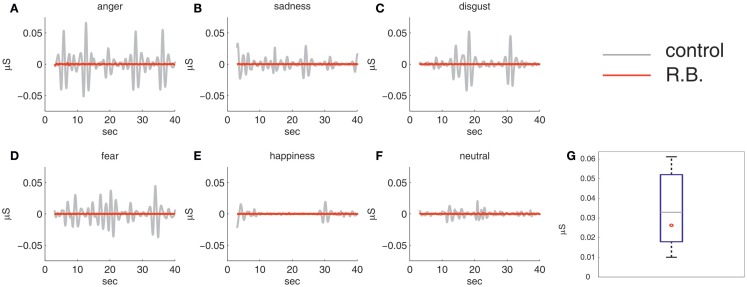
**Results of physiological reactivity: R.B.’s electrodermal activity during the film clips set (A–F) against a prototypical electrodermal response curve of a healthy control subject**. Comparing R.B.’s skin conductance during the respiration measurement with the control group in terms of averaged trough-to-peak distances showed no significant difference, as displayed in the box-plot diagram **(G)**.

On the level of raw scores, R.B. developed no SC responses to any kind of film material presented to him (Table 4), although he had passed the respiration test in the beginning of the experiment without any observable differences to the control group (*z*_respiration_ = −0.46). Thus, R.B.’s arousal level appeared not to be affected by any experimental manipulation of emotional stimulation. The control group showed more physiological fluctuations subject to the different emotional content of the stimulus material. These findings are illustrated in Figure 3 by plotting R.B.’s response curve in contrast to a prototypical response curve of a healthy control subject for all experimental conditions applied. Results of the respiration test are included additionally.

**Table 4:** **Data of physiological reactivity (nSCR)**.

Emotion in the film	R.B.		Control group
	nSCR	*z*-Score	nSCR
Anger	0	−1.02	*M* = 6.81, SD = 6.69, range 0–20
Sadness	0	−0.69	*M* = 3.50, SD = 5.05, range 0–16
Disgust	0	−1.03	*M* = 6.00, SD = 5.82, range 0–16
Fear	0*	−2.16*	*M* = 14.56, SD = 6.73, range 3–23
Happiness	0	−0.79	*M* = 4.31, SD = 5.42, range 0–15
Neutral	0	−0.78	*M* = 3.19, SD = 4.10, range 0–12

**M*, mean; SD, standard deviation; nSCR, number of skin conductance responses*.

*Significantly abnormal values (i.e., |*z*| ≥ 1.96) are marked by an asterisk*.

Relating the number of SCR observed in R.B. to control group means, our analyses revealed no significant differences in case of anger (*z*_anger_ = −1.02), sadness (*z*_sadness_ = −0.69), disgust (*z*_disgust_ = −1.03), happiness (*z*_happiness_ = −0.79), or neutral film material (*z*_neutral_ = −0.78) due to high variance among the healthy controls (Table 4). However, a significant deviation was registered for the fear-evoking film clip (*z*_fear_ = −2.16). Only in this condition, all healthy control subjects developed SCRs (*M* = 14.56, SD = 6.73, range 3–23 nSCR), whereas in other conditions, there were between four and eight subjects without noticeable SCRs.

## Discussion

The subject of the present study was to examine behavioral and autonomic responses to emotional film sequences in a patient suffering from early adaptive bilateral amygdala (and anterior hippocampal) inflammation due to LE with CASPR2-antibodies in comparison to a healthy control sample. During the presentation of six discrete emotional video clips autonomic measures (i.e., SCRs) as well as individual reports of emotion, arousal and valence were collected.

In accordance with our hypothesis, the patient demonstrated the absence of SCRs to all stimuli presented, which was in marked contrast to healthy controls. Inspection of the SCR profiles revealed a nearly complete lack of SC changes during the emotional visual stimulation in the patient regardless of the film clip shown. This deficit, however, was not due to a general problem in the generation of autonomic responses (e.g., a potential autonomic neuropathy due to R.B.’s diabetes mellitus), since the patient was able to show these responses during a deep respiration test, without differing from the control group (Figure 3). Rather, results reveal the absence of emotion-provoked autonomic modulations of SCRs, which seems to be due to the pronounced amygdala involvement in LE. The amygdala is known to modulate autonomic responses due to emotional stimulation. Thus, our results are in line with studies reporting a significant decrease of SCRs in subjects with amygdala lesions processing emotionally arousing pictures (23) or in patients with LE being exposed to arousing task demands (24, 25). In both studies, patients showed impaired emotional modulation of SC. Even though LE does not only affect the amygdala, these findings are in accordance with our proposal that specifically the debilitated function of the amygdala is responsible for the impaired modulation of the SCRs in our patient. This is corroborated by the fact that the amygdala shows mostly pronounced pathological changes in R.B., as revealed by neuroimaging methods applied.

A statistically significant difference between the patient and the control group was given only in case of the fear-evoking film clip. Whereas other video sequences could not evoke SCRs in all control subjects because they were presumably not intensive enough for everyone, the fear-inducing film clip created constant suspense and a different kind of relevance by showing a person exposed to enduring mortal danger, thus determining clear autonomic responses to that scene in all normal subjects. As the patient did not show any autonomic activation, we see the effect of emotional stimulation in LE here most clearly. The fear-inducing film clip provided highest discriminative power due to its exceptional intensity and stimulating quality.

Remarkably, arousal and valence ratings did not differ between R.B. and controls for fearful film clips in our study. There were only differences in valence ratings for the funny video clip and in arousal ratings for the neutral video clip. Additionally, the patient was impaired in correctly judging the basic emotions of happiness and disgust. In line with this result, multiple case studies of patients with amygdalar damage reported relatively preserved social and emotional behavior (8), which may be related to successful cognitive compensations for the loss of emotional functions. Thus, unharmed understanding of social and emotional behavior as well as an intact subjective discernment of emotion might be enough to guide emotional behavior in most situations, especially if the personal development took place with an intact amygdala (16, 17), as this was also the case with R.B. However, it remains to be investigated in future studies, whether patients with LE show indeed a dissociation between behavioral data and autonomic responses for different classes of emotional stimuli. Sprengelmeyer et al. (15) demonstrated an emotion recognition deficit in a single case suffering from the paraneoplastic form of LE when recognizing fear and disgust from both faces and voices, suggesting that at least some patients might exhibit emotion recognition deficits.

It should be mentioned that our findings have to be interpreted with caution, since there are limitations in the study. First, the control group was not perfectly matched in terms of age or educational level. Furthermore, the patient but none of the control subjects suffered from diabetes mellitus. However, potential influences of clinical status and age would have been manifest during the respiration measurement; this was not the case, as we found no differences between R.B. and the control group. As for educational level, the experimental task did not pose high cognitive demands and is commonly used in multiple clinical samples for investigations of basal emotional processing (11). Thus, it is reasonable to assume that the control group was suitable for statistical comparisons and the observed effect of R.B.’s non-responsiveness during emotional stimulation is a true one. Second, we investigated only a single case, a limitation dictated by the rareness of patients with this diagnosis. Future studies are strongly needed, in which samples of patients with LE are systematically investigated with different emotional tasks and dependent measures in order to explore the basic emotional disturbances in patients suffering from LE in more detail. Nevertheless, the current findings add to the literature a clear example of a pronounced autonomic deficit in LE, especially in response to fear-inducing stimuli, which is already present early in the course of the disease.

## Author Contributions

JK, NM, and TS designed the study. JK prepared the experiment and the technical settings. ES and OS performed the data collection, the statistical analyses, and wrote the first draft of the article. MB prepared the physiological data. HL performed the clinical neuropsychological examination. KG and NM recruited the patient, NM and HW provided expert advice on limbic encephalitis and the FLAIR images together with WS. All authors contributed to and approved the final manuscript.

## Conflict of Interest Statement

The authors declare that the research was conducted in the absence of any commercial or financial relationships that could be construed as a potential conflict of interest.
